# Multiplatform-Integrated Identification of Melatonin Targets for a Triad of Psychosocial-Sleep/Circadian-Cardiometabolic Disorders

**DOI:** 10.3390/ijms24010860

**Published:** 2023-01-03

**Authors:** Luciana Aparecida Campos, Ovidiu Constantin Baltatu, Sergio Senar, Rym Ghimouz, Eman Alefishat, José Cipolla-Neto

**Affiliations:** 1 Center of Innovation, Technology, and Education (CITE) at Anhembi Morumbi University—Anima Institute, Sao Jose dos Campos Technology Park, Sao Jose dos Campos 12247-016, Brazil; 2 Department of Public Health and Epidemiology, College of Medicine and Health Science, Khalifa University, Abu Dhabi P.O. Box 127788, United Arab Emirates; 3 DrTarget, 28806 Madrid, Spain; 4 Fatima College of Health Sciences, Abu Dhabi P.O. Box 3798, United Arab Emirates; 5 Department of Pharmacology, College of Medicine and Health Science, Khalifa University, Abu Dhabi P.O. Box 127788, United Arab Emirates; 6 Department of Biopharmaceutics and Clinical Pharmacy, Faculty of Pharmacy, The University of Jordan, Amman 11942, Jordan; 7 Center for Biotechnology, Khalifa University, Abu Dhabi P.O. Box 127788, United Arab Emirates; 8 Department of Physiology and Biophysics, Institute of Biomedical Sciences, University of São Paulo, São Paulo 05508-000, Brazil

**Keywords:** biomarkers, psychological stress, sleep/circadian rhythm, cardiovascular disorders, machine learning

## Abstract

Several psychosocial, sleep/circadian, and cardiometabolic disorders have intricately interconnected pathologies involving melatonin disruption. Therefore, we hypothesize that melatonin could be a therapeutic target for treating potential comorbid diseases associated with this triad of psychosocial-sleep/circadian-cardiometabolic disorders. We investigated melatonin’s target prediction and tractability for this triad of disorders. The melatonin’s target prediction for the proposed psychosocial-sleep/circadian-cardiometabolic disorder triad was investigated using databases from Europe PMC, ChEMBL, Open Targets Genetics, Phenodigm, and PheWAS. The association scores for melatonin receptors MT1 and MT2 with this disorder triad were explored for evidence of target–disease predictions. The potential of melatonin as a tractable target in managing the disorder triad was investigated using supervised machine learning to identify melatonin activities in cardiovascular, neuronal, and metabolic assays at the cell, tissue, and organism levels in a curated ChEMBL database. Target–disease visualization was done by graphs created using “igraph” library-based scripts and displayed using the Gephi ForceAtlas algorithm. The combined Europe PMC (data type: text mining), ChEMBL (data type: drugs), Open Targets Genetics Portal (data type: genetic associations), PhenoDigm (data type: animal models), and PheWAS (data type: genetic associations) databases yielded types and varying levels of evidence for melatonin-disease triad correlations. Of the investigated databases, 235 association scores of melatonin receptors with the targeted diseases were greater than 0.2; to classify the evidence per disease class: 37% listed psychosocial disorders, 9% sleep/circadian disorders, and 54% cardiometabolic disorders. Using supervised machine learning, 546 cardiovascular, neuronal, or metabolic experimental assays with predicted or measured melatonin activity scores were identified in the ChEMBL curated database. Of 248 registered trials, 144 phase I to IV trials for melatonin or agonists have been completed, of which 33.3% were for psychosocial disorders, 59.7% were for sleep/circadian disorders, and 6.9% were for cardiometabolic disorders. Melatonin’s druggability was evidenced by evaluating target prediction and tractability for the triad of psychosocial-sleep/circadian-cardiometabolic disorders. While melatonin research and development in sleep/circadian and psychosocial disorders is more advanced, as evidenced by melatonin association scores, substantial evidence on melatonin discovery in cardiovascular and metabolic disorders supports continued R&D in cardiometabolic disorders, as evidenced by melatonin activity scores. A multiplatform analysis provided an integrative assessment of the target–disease investigations that may justify further translational research.

## 1. Introduction

**Figure 1:**
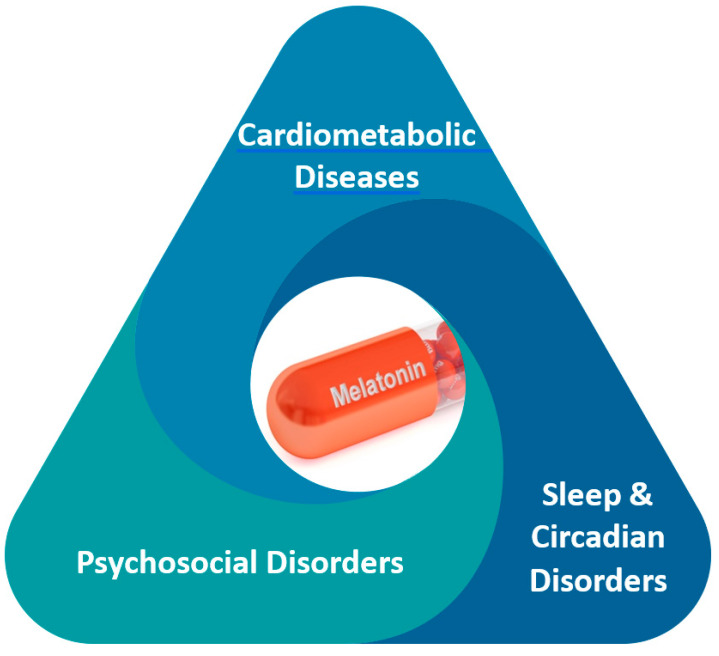
Melatonin and its related drugs as potential treatments for the psychosocial-sleep/circadian-cardiometabolic disorder triad.

Melatonin and circadian systems are responsible for optimizing our organism’s physiology in preparation for the anticipated exposure to different mental and physical demands during the day by maintaining a regular schedule of resting, recuperating, repairing, and preparing for the day during the night [1]. Melatonin is a hormone marketed as a dietary supplement and frequently prescribed for insomnia [2]. Only recently, the FDA proposed melatonin for inclusion on the list of bulk drug substances for use in compounding under section 503A of the Federal Food, Drug, and Cosmetic Act (FD&C Act) and for the treatment of sleep disorders [3]. Currently, proof-of-concept clinical trials are being conducted to investigate the druggability potential of melatonin and its related drugs for several disorders, which will ideally result in a more regulated application of this hormone [4]. Evidence demonstrating melatonin’s ability to regulate the circadian clock and sleep has led to translational research on the potential of melatonin as a treatment for human disease, particularly circadian rhythm and sleep disorders, and psychosocial disorders [5,6], with other implications as an adjuvant in cardiovascular and metabolic diseases [7]. Cardiometabolic diseases are a group of maladaptive cardiovascular, renal, thrombotic, inflammatory, and metabolic diseases that have long been recognized by international health organizations [8,9]. It has been demonstrated that cardiovascular and metabolic processes are not only influenced by the behavioral sleep/wake cycle but are also under the direct control of melatonin (the circadian system’s nighttime signal) and the master circadian pacemaker located in the suprachiasmatic nucleus [10]. Circadian and sleep disorders can cause melatonin suppression, as well as effects on other physiological variables such as heart rate, cortisol, and temperature during the biological night, which can have a negative impact on cardiometabolic regulation [10]. The interrelationship between circadian, cardiometabolic, neurologic, and psychiatric disorders has hinted at potential circadian-based therapeutic strategies, such as exogenous melatonin and chronotherapy, to improve disease outcomes (Figure 1) [11].

Cardiometabolic and sleep/circadian disorders have intricately interconnected pathologies (Figure 1). Sleep and circadian disorders have long been recognized for their detrimental effects on cardiometabolic, neurologic, psychiatric, and immune disorders [11,12]. There is growing evidence that sleep and circadian disorders are related to cardiometabolic diseases and cardiovascular risk factors (reviewed by Thosar et al. [13] and Crnko et al. [14]). Poor objective sleep efficiency, long wake-up after sleep onset, and daytime sleepiness were associated with an increased risk of incident cardiometabolic disease [15,16,17,18]. Patients with circadian blood pressure disorders, such as “non-dippers” (no physiological decline in nighttime blood pressure) or “reverse dippers” (increase in nocturnal blood pressure above day levels), are at risk of cardiovascular events and mortality [19,20,21].

Cardiometabolic and psychosocial disorders have intricately related pathologies (Figure 1). The mind-body connection has been extensively examined in cardiovascular diseases (CVD) [22], revealing a bidirectional link between cardiovascular diseases and psychological disorders [23]. Several systematic analyzes have shown that psychosocial stress is a critical determinant of cardiovascular disease and mortality [24]. Anxiety and depression are often comorbid [25] and are identified as psychosocial risk factors for cardiovascular disease [26,27]. Psycho-cardiological disease has been defined as a multidisciplinary condition that includes psychology, psychiatry, clinical medicine (including the cardiovascular system and neurology), public health, environmental health, and occupational health [28]. Both the European Society of Cardiology and the American Heart Association have stated in position papers that depression may be a modifiable risk factor for coronary heart disease (CHD), urging greater detection and care of the condition [29,30].

Sleep/circadian and psychosocial disorders have intricately interconnected pathologies (Figure 1). The stress system is intrinsically related to the circadian clock system, and the dysfunction of the former causes dysregulation of the latter, and vice versa [31]. Circadian dysregulation after stress exposure (such as posttraumatic chronodisruption) may be a key feature of stress-related diseases, leading to pathological manifestations of traumatic stress through disruption of temporal order at different organizational levels [32]. As a consequence of psychological stress, anxiety and depression produce a cascade of pathological responses that implicate melatonin and cause sleep abnormalities [33,34] and circadian rhythm disturbances [6,35]. In contrast, several types of sleep disorders and non-sleep circadian disorders have been proven to be risk factors for future depression [36]. Sleep disturbances are among the symptoms listed in the diagnostic criteria of DSM-5 for a major depressive episode, along with weight/appetite changes, psychomotor agitation or retardation, fatigue, worthlessness or guilt, and executive dysfunction [37,38].

Ongoing research is developing evidence that the circadian system is associated with cardiovascular metabolic diseases and psychosocial disorders, which often are encountered as comorbidities [39]. Melatonin is believed to be a key modulator in the molecular pathways that relate circadian/sleep disorders to psychosocial disorders such as anxiety and depression [40]. Moreover, melatonin is being investigated as a therapeutic target for cardiovascular and metabolic diseases [4]. To summarize, several psychosocial, sleep/circadian, and cardiometabolic disorders have intricately interconnected pathologies that involve the disruption of melatonin. Therefore, we hypothesize that melatonin has the potential to be a therapeutic target for the treatment of possible comorbid diseases related to the psychosocial-sleep/circadian-cardiometabolic triad (Figure 1) [41]. The aim of this study was to evaluate the target prediction and tractability of melatonin for the psychosocial-sleep/circadian-cardiometabolic disease triad using integrated research on open-access databases.

## 2. Results

### 2.1. Target Prediction for Melatonin in Disorders of the Psychosocial-Sleep/Circadian-Cardiometabolic Triad

**Figure 2:**
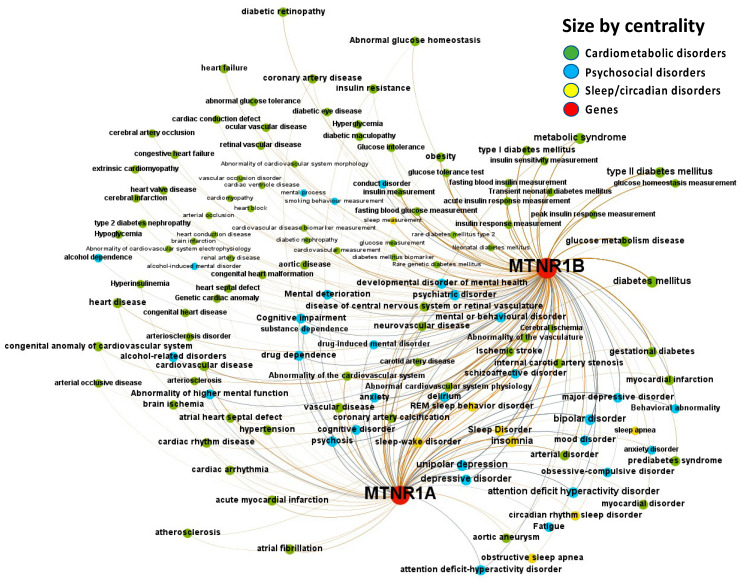
Knowledge Network visualization graph combining heterogeneous information and metadata from different databases. Presented are associations (edges, edge color is a combination of the colors of the connected nodes) between melatonin receptors MT1 (MTNR1A) and MT2 (MTNR1B) (red nodes) and the investigated disease triad (nodes color-coded per group of disorders in the triad) converged from the Europe PMC, ChEMBL, Open Targets Genetics, Phenodigm, and PheWAS databases. Edges width is the result of the addition of all association scores connecting each pair of nodes. Similarly, nodes have been sized by their respective weighed degree, i.e., the total sum of association scores corresponding to all edges connecting each node with any other, following a logarithmic scale to provide better visualization of size differences between disease nodes. Finally, the application of a ForceAtlas algorithm results in a distribution where proximity between nodes is proportional to the strength of their association.

The Europe PMC, ChEMBL, Open Targets Genetics, Phenodigm, and PheWAS databases were utilized to examine melatonin’s target prediction for the psychosocial-sleep/circadian-cardiometabolic disorder triad. Figure 2 presents the associations between melatonin and target diseases from the combined analysis of the five database sources. The logic behind incorporating results from these data sources is that, when a relationship is supported by clinical trials, the literature, animal models, and genetic research, the disease node has a larger size than another that is just supported by reports from the literature. When an association between two nodes contains parallel edges, the number of connections of the node grows, increasing node size. The node’s size is determined by its degree, which is the number of edges connecting each node to the network. Several diseases belonging to the psychosocial-sleep/circadian-cardiometabolic disorder triad have been related to melatonin as a potential treatment target, as illustrated by the densely-filled graph with nodes of varying magnitudes (446 associations were found between melatonin and the targeted diseases, of which 235 [52.7%] had data source scores greater than 0.2 to classify the evidence per disease class). Numeric values for these scores, together with the evidence count, disease label, target name, association type (direct target–disease associations incorporated from data sources, as well as ‘indirect’ associations where evidence is applied throughout the ontological structure of disease classification) and disease group, are presented as a supplementary MS Excel table (File S1: Excel sheet “AssociationScoresByDataSource”).

**Figure 3:**
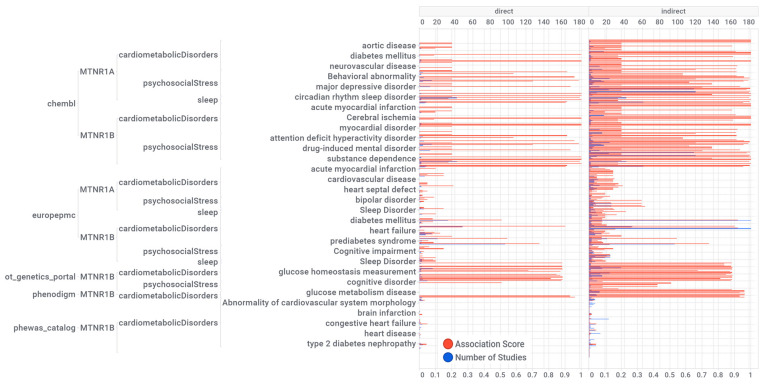
Knowledge visualization brace mapping for melatonin receptors MT1 (MTNR1A) and MT2 (MTNR1B) associations and evidence with diseases by the data source. The numerical values are presented in the supplementary material. The units at the top and bottom of the horizontal bar graphs are the number of studies and association scores, respectively. Numeric values, disease label, target name, association type (direct or indirect), and disease group are reported in a supplementary MS Excel table (File S1: Excel sheet “AssociationScoresByDataSource”).

The mapping of the knowledge visualization brace in Figure 3 details the associations of melatonin with the triad of psychosocial-sleep/circadian-cardiometabolic disorders grouped by source of knowledge, pharmaceutical target, and a class of diseases with direct or indirect disease association scores. Numeric values for these scores, together with the evidence count, disease label, target name, association type (direct or indirect), and disease group, are presented as a supplementary MS Excel table (File S1: Excel sheet “AssociationScoresByDataSource”). High association scores (higher than 0.2) were found for psychosocial disorders (37%): schizoaffective disorder, unipolar depression, major depressive disorder, depressive disorder, anxiety disorder, bipolar disorder, psychosis, delirium, attention deficit hyperactivity disorder, obsessive-compulsive disorder, fatigue, drug dependence, drug-induced mental disorder, substance dependence, alcohol-related disorders, mental or behavioral disorder, mood disorder, cognitive impairment, and developmental disorder of mental health; sleep/circadian disorders (9%): REM sleep behavior disorder, sleep disorder, insomnia, sleep-wake disorder, and circadian rhythm sleep disorder; and cardiometabolic disorders (54%): internal carotid artery stenosis, carotid artery disease, coronary artery calcification, ischemic stroke, cerebral ischemia, neurovascular disease, vascular disease, aortic aneurysm, disease of central nervous system or retinal vasculature, hypertension, atrial fibrillation, cardiac rhythm disease, acute myocardial infarction, gestational diabetes, rare diabetes mellitus type 2, diabetes mellitus, glucose metabolism disease, type I diabetes mellitus, prediabetes syndrome, transient neonatal diabetes mellitus, glucose intolerance, glucose metabolism disease, metabolic syndrome, obesity.

**Figure 4:**
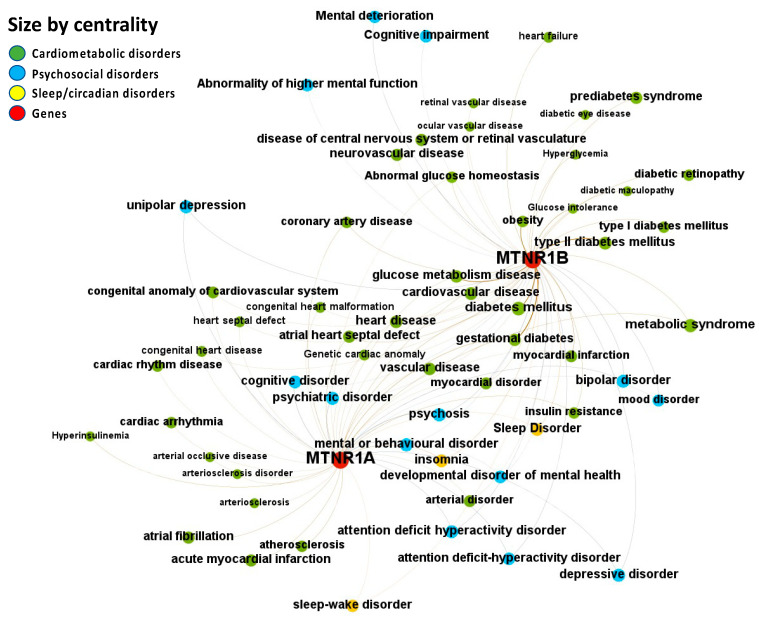
Node-Weighted Network graph—Europe PMC database. Presented are associations (edges, edge color is a combination of the colors of the connected nodes) between melatonin receptors MT1 (MTNR1A) and MT2 (MTNR1B) (red nodes) and the investigated disease triad (nodes color-coded per group of disorders in the triad) from the Europe PMC database. Edges width is the result of the addition of all association scores connecting each pair of nodes. Similarly, nodes have been sized by their respective weighed degree, i.e., the total sum of association scores corresponding to all edges connecting each node with any other, following a logarithmic scale to provide better visualization of size differences between disease nodes. Finally, the application of a ForceAtlas algorithm results in a distribution where proximity between nodes is proportional to the strength of their association.

**Figure 5:**
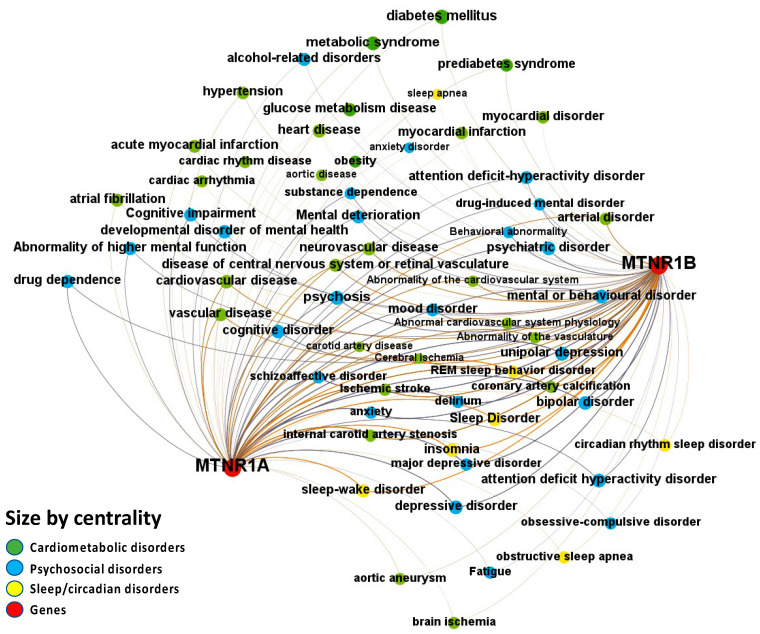
Node-Weighted Network graph—ChEMBL database. Presented are associations (edges, edge color is a combination of the colors of the connected nodes) between melatonin receptors MT1 (MTNR1A) and MT2 (MTNR1B) (red nodes) and the investigated disease triad (nodes color-coded per group of disorders in the triad) from the ChEMBL database. Edges width is the result of the addition of all association scores connecting each pair of nodes. Similarly, nodes have been sized by their respective weighed degree, i.e., the total sum of association scores corresponding to all edges connecting each node with any other, following a logarithmic scale to provide better visualization of size differences between disease nodes. Finally, the application of a ForceAtlas algorithm results in a distribution where proximity between nodes is proportional to the strength of their association.

**Figure 6:**
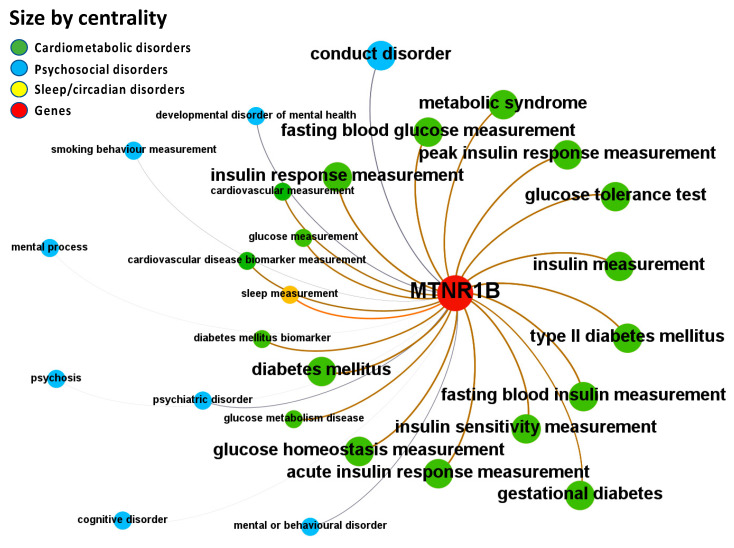
Node-Weighted Network graph—Open Targets Genetics Portal. Presented are associations (edges, edge color is a combination of the colors of the connected nodes) between melatonin receptor MT2 (MTNR1B) (red nodes) and the investigated disease triad (nodes color-coded per group of disorders in the triad) from the Open Targets Genetics Portal. Edges width is the result of the addition of all association scores connecting each pair of nodes. Similarly, nodes have been sized by their respective weighed degree, i.e., the total sum of association scores corresponding to all edges connecting each node with any other, following a logarithmic scale to provide better visualization of size differences between disease nodes. Finally, the application of a ForceAtlas algorithm results in a distribution where proximity between nodes is proportional to the strength of their association. No data regarding MT1 (MTNR1A) were identified in the database.

**Figure 7:**
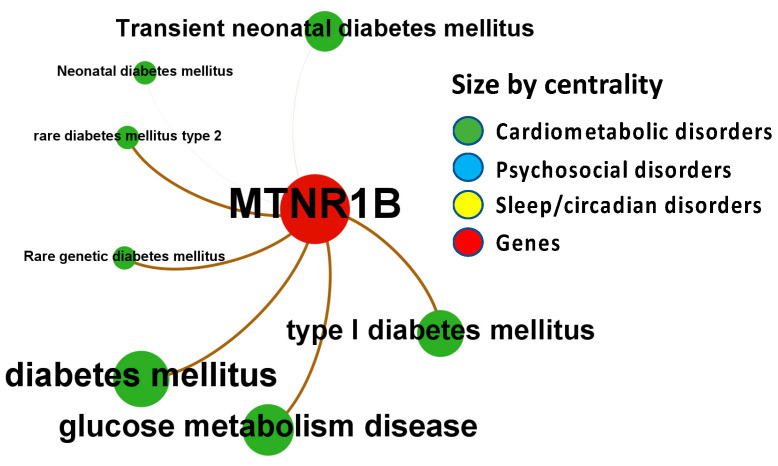
Node-Weighted Network graph—Phenodigm. Presented are associations (edges, edge color is a combination of the colors of the connected nodes) between melatonin receptor MT2 (MTNR1B) (red nodes) and the investigated disease triad (nodes color-coded per group of disorders in the triad) from the Phenodigm. Edges width is the result of the addition of all association scores connecting each pair of nodes. Similarly, nodes have been sized by their respective weighed degree, i.e., the total sum of association scores corresponding to all edges connecting each node with any other, following a logarithmic scale to provide better visualization of size differences between disease nodes. Finally, the application of a ForceAtlas algorithm results in a distribution where proximity between nodes is proportional to the strength of their association. No data regarding MT1 (MTNR1A) were identified in the database.

**Figure 8:**
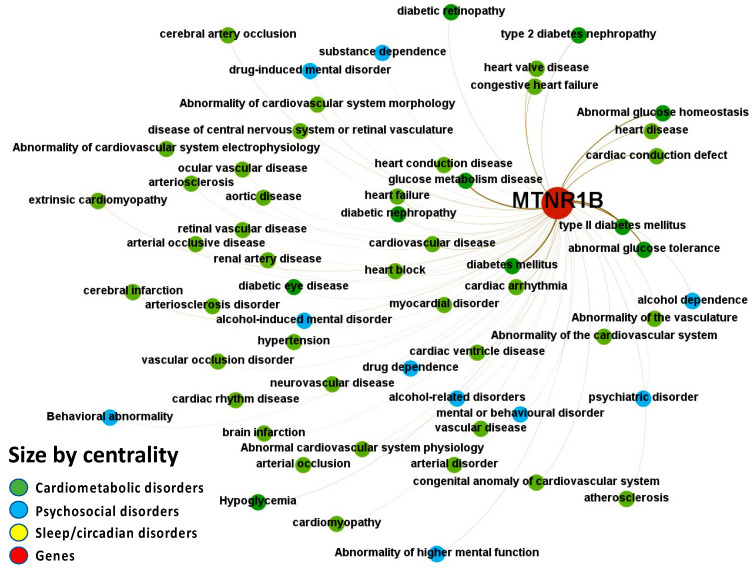
Node-Weighted Network graph—PheWAS. Presented are associations (edges, edge color is a combination of the colors of the connected nodes) between melatonin receptor MT2 (MTNR1B) (red nodes) and the investigated disease triad (nodes color-coded per group of disorders in the triad) from the phenome-wide association studies (PheWAS) portal. Edges width is the result of the addition of all association scores connecting each pair of nodes. Similarly, nodes have been sized by their respective weighed degree, i.e., the total sum of association scores corresponding to all edges connecting each node with any other, following a logarithmic scale to provide better visualization of size differences between disease nodes. Finally, the application of a ForceAtlas algorithm results in a distribution where proximity between nodes is proportional to the strength of their association. No data regarding MT1 (MTNR1A) were identified in the database.

Melatonin associations with diseases, which were corroborated by different levels of evidence from different data sources, are depicted in Figure 4, Figure 5, Figure 6, Figure 7 and Figure 8.

Melatonin-disease co-occurrences were discovered in the Europe PMC database using deep-learning-based Named Entity Recognition (NER). Melatonin participation in the psychosocial-sleep/circadian-cardiometabolic disorder triad has been linked to receptors of type MT1 (MTNR1A) and MT2 (MTNR1B) (Figure 4 and File S1: Excel Sheet “OpTarChemblAssociationScoresCli”). High association scores (higher than 0.2) were found for psychosocial disorders: psychosis, cognitive disorder, and mental or behavioral disorder; and cardiometabolic disorders: gestational diabetes, type II diabetes mellitus, obesity, glucose intolerance, and myocardial infarction. The literature references for the melatonin associations can be found in File S1, Excel sheet “literatureEvidencesEuropePMC”.

The ChEMBL database reports the melatonin receptors MT1 (MTNR1A) and MT2 (MTNR1B) as targets for the psychosocial-sleep/circadian-cardiometabolic disorder triad (Figure 5 and File S1: Excel sheet “OpTarChemblAssociationScoresCli”). High association scores (higher than 0.2) were found for psychosocial disorders: depression, anxiety, schizoaffective disorder, psychosis, delirium, mood disorder, bipolar disorder, depressive disorder, anxiety disorder, drug-induced mental disorder, substance dependence, cognitive impairment, attention deficit hyperactivity disorder, obsessive-compulsive disorder, fatigue, and alcohol-related disorders; sleep/circadian disorders: REM sleep behavior disorder, sleep disorder, insomnia, sleep-wake disorder, and circadian rhythm sleep disorder; and cardiometabolic disorders: internal carotid artery stenosis, carotid artery disease, coronary artery calcification, ischemic stroke, cerebral ischemia, hypertension, heart disease, metabolic syndrome, atrial fibrillation, aortic aneurysm, prediabetes syndrome, myocardial infarction, obesity, and myocardial disorder. Investigational and approved indications for melatonin curated from clinical trial records and post-marketing package inserts included melatonin, agomelatine, ramelteon, and tasimelteon. To date, of 248 registered trials, 144 phase I to IV trials for melatonin or agonists have been completed, and 78 phase IV trials are ongoing or complete for drugs with investigational or approved indications targeting melatonin receptors based on their proposed mode of action on the psychosocial-sleep/circadian-cardiometabolic disorder triad. Of 144 completed trials, 33.3% are for psychosocial disorders, 59.7% are for sleep/circadian disorders, and 6.9% are for cardiometabolic disorders (File S1: Excel sheet “OpTarChemblAssociationScoresCli”).

Melatonin-related evidence for MT2 (MTNR1B) was identified, integrated, and summarized using the Locus2Gene approach in Open Targets. The most common diseases associated with the melatonin MT2 receptor, as revealed through the investigation of the Open Targets Genetics portal, are metabolic and psychosocial disorders (Figure 6 and File S1: Excel sheet “OpTarChemblAssociationScoresCli”). High association scores (higher than 0.2) were found for psychosocial disorders: conduct disorder, mental or behavioral disorder, psychiatric disorder, and developmental disorder of mental health; sleep/circadian disorders: sleep diseases; and cardiometabolic disorders: diabetes mellitus, type II diabetes mellitus, gestational diabetes, cardiovascular diseases, and metabolic syndrome.

To discover melatonin-related phenotypes and to connect clinical aspects reported in humans to mouse phenotype annotations, we use the PhenoDigm algorithm, which prioritizes disease-causing genes based on phenotype information. Figure 7 illustrates the associations between melatonin receptor MT2 (MTNR1B) with disorders of the psychosocial-sleep/circadian-cardiometabolic disorder triad (data presented in File S1: Excel sheet “OpTarChemblAssociationScoresCli”). High association scores (higher than 0.2) were found for psychosocial disorders: developmental disorder of mental health, mental or behavioral disorder, psychiatric disorder, cognitive disorder, and psychosis; and cardiometabolic disorders: cardiovascular cancer, vascular anomaly, cardiovascular neoplasm, cardiovascular disease, diabetes mellitus, rare diabetes mellitus type 2, type I diabetes mellitus, cerebral small vessel disease, disease of central nervous system or retinal vasculature, neurovascular disease, and transient neonatal diabetes mellitus.

Melatonin-associated phenotypes were retrieved from an EHR database using the PheWAS (phenome-wide association studies) method, and modifications were made to convert raw EHR data to designated cases and controls for analysis to assist in determining disease subtypes. Figure 8 illustrates the associations identified between melatonin receptor MT2 (MTNR1B) and disorders from the psychosocial-sleep/circadian-cardiometabolic disorder triad (data presented in File S1: Excel sheet “OpTarChemblAssociationScoresCli”). PheWAS association scores were all lower than 0.2 for psychosocial disorders: alcohol dependence, drug dependence, mental or behavioral disorder, drug-induced mental disorder, psychiatric disorder, and abnormality of higher mental function; and cardiometabolic disorders: diabetes mellitus, glucose metabolism disease, type II diabetes mellitus, abnormal glucose tolerance, cardiac conduction defect, heart disease, cardiovascular disease, cardiac arrhythmia, congestive heart failure, heart failure, heart valve disease, heart conduction disease, heart block, myocardial disorder, cardiac ventricle disease, diabetic nephropathy, diabetic retinopathy, ocular vascular disease, vascular disease, disease of central nervous system or retinal vasculature, neurovascular disease, hypertension, aortic disease, renal artery disease, abnormality of cardiovascular system electrophysiology, congenital anomaly of cardiovascular system, cardiac rhythm disease, atherosclerosis, arterial occlusive disease, cardiomyopathy, cerebral artery occlusion, cerebral infarction, and hypoglycemia.

**Figure 9:**
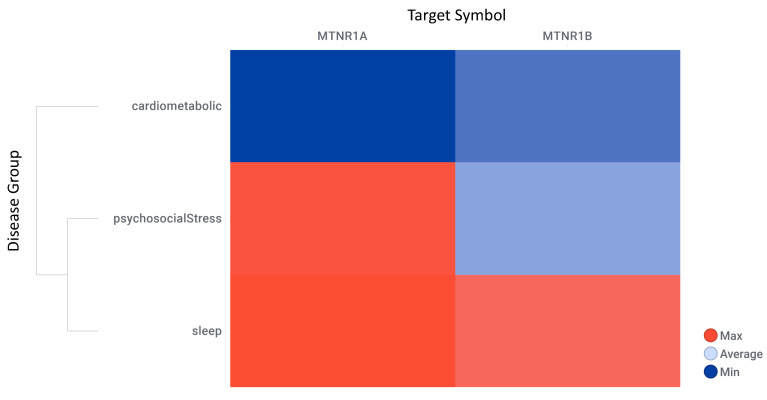
OpenTargets harmonic scores hierarchical clustering by disease group and melatonin receptors. Target symbol: melatonin receptors MT1 (MTNR1A) and MT2 (MTNR1B).

OpenTargets harmonic scores hierarchical clustering by disease group and melatonin receptors shows that research is more advanced for MT1 (MTNR1A) in sleep disorders, while cardiometabolic studies for melatonin receptors are less advanced (Figure 9, detailed data on File S1: Excel sheet “AssociationScoresByDataSource”, column “Avg (datasourceHarmonicScore) for superDiseaseGroup”. The mean rank differences between the averaged harmonic scores of the investigated disease groups are (Dunn’s multiple comparisons test) −205.0 for “Cardiometabolic Diseases vs. Psychosocial Disorders”, −291.5 for “Cardiometabolic Diseases vs. Sleep & Circadian Disorders”, and −86.50 for “Psychosocial Disorders vs. Sleep & Circadian Disorders”, with significance by adjusted *p* value of <0.0001 for all comparisons.

**Figure 10:**
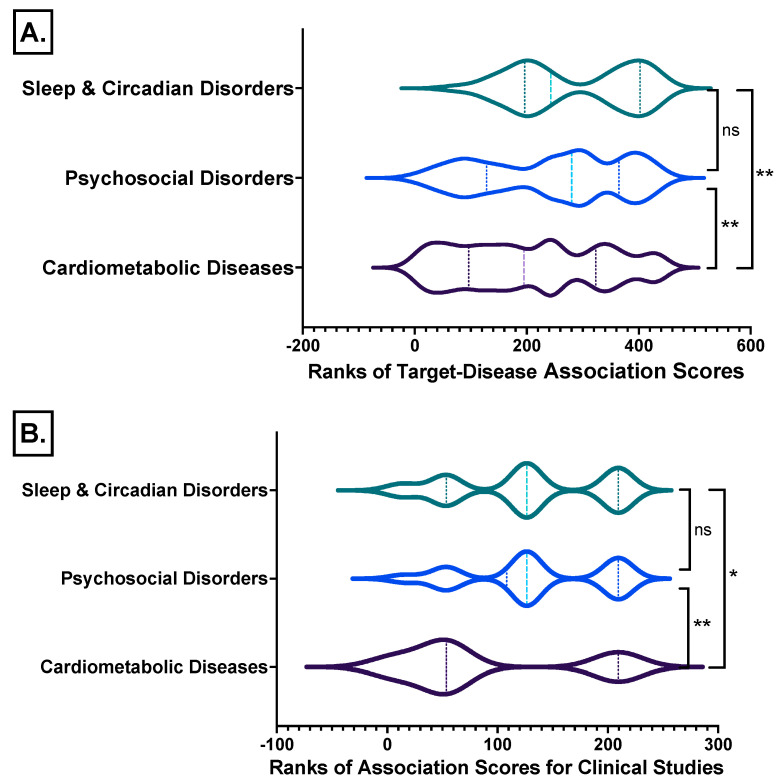
Ranks comparisons of the target–disease association scores of the disease triad groups studied for the melatonin receptors MT1 (MTNR1A) and MT2 (MTNR1B) from all databases analyzed from (**A**) all data identified and from (**B**) clinical studies. *, Kruskal-Wallis test with Dunn’s correction; **, *p* ≤ 0.01; ns, not significant differences between groups. Detailed data File S1: Excel sheet “AssociationScoresByDataSource”, column “Avg (datasourceHarmonicScore) for superDiseaseGroup”.

The target–disease association scores of the examined disease triad groups for the melatonin receptors MT1 (MTNR1A) and MT2 (MTNR1B) show that melatonin receptor research in sleep/circadian and psychosocial disorders is significantly more advanced than in cardiometabolic disorders (Figure 10A; File S1: Excel sheet “AssociationScoresByDataSource”), which applies for clinical studies as well (Figure 10B; File S1: Excel sheet “OpTarChemblAssociationScoresCli”).

### 2.2. Melatonin Target Tractability Using Supervised Machine Learning

**Figure 11:**
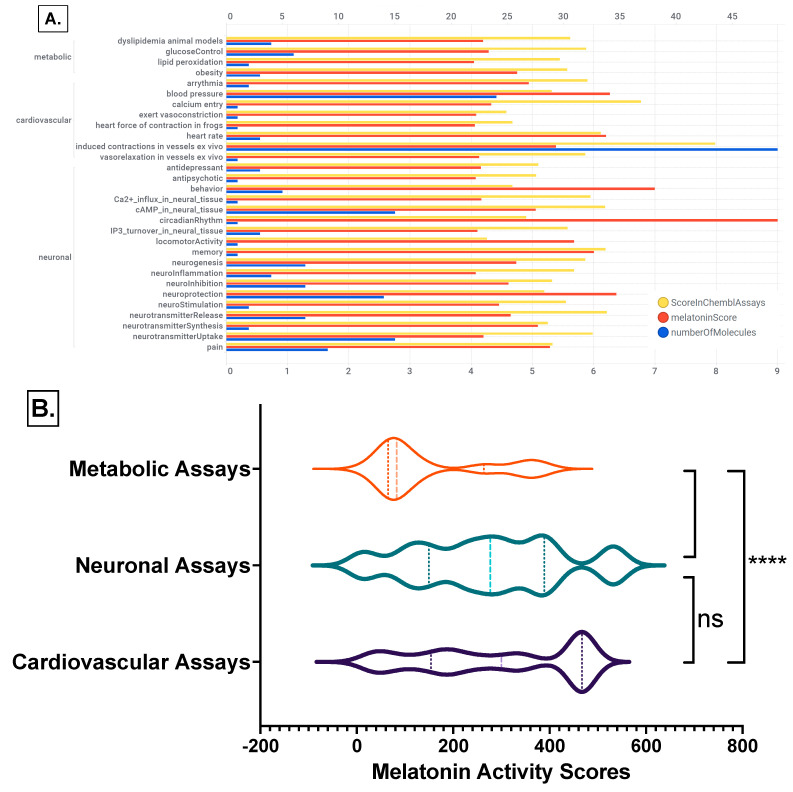
Melatonin activities on ChEMBL cardiovascular, neuronal, or metabolic experimental assays. (**A**) Effect of melatonin actives on ChEMBL assays bar chart by assay category and biological system; (**B**) ranks comparisons of the melatonin activity scores per biological assays categories: Kruskal-Wallis test with Dunn’s correction; ****, *p* < 0.0001; ns, not significant differences between groups. Detailed data File S1: Excel sheet “MLforMELactivityScores”.

Melatonin target tractability was explored using melatonin activity scores, which provide an assessment of the likelihood of identifying a modulator that interacts effectively with the target or pathway. The melatonin activity scores were categorized per cardiovascular, neuronal, or metabolic experimental assays as the biological systems mainly involved in the psychosocial-sleep/circadian-cardiometabolic disorders triad. We retrieved 546 records with cardiovascular, neuronal, or metabolic experimental assays having melatonin activity scores greater than 4, actual or predicted, and with chemblActivityScores more than 4.5 (Figure 11, and Supplementary File S1, “MLforMELactivityScores” Excel sheet).

The mean rank differences between the melatonin activity scores between the investigated ChEMBL assay categories are (Dunn’s multiple comparisons test) −142.9 **** for “Metabolic Assays vs. Cardiovascular Assays”, −124.8 **** for “Metabolic Assays vs. Neuronal Assays”, and 18.18 ^ns^ for “Cardiovascular Assays vs. Neuronal Assays”, with significance by adjusted *p* value of <0.0001 for all comparisons (Figure 11B).

## 3. Discussion

The main findings of this study are the target prediction and tractability of melatonin receptors for the proposed psychosocial-sleep/circadian-cardiometabolic disease triad, which supports melatonin’s druggability potential. The target–disease prediction for the melatonin receptors based on the association scores of the examined disease triad groups demonstrates that research on melatonin receptors in sleep/circadian and psychosocial disorders is significantly more advanced than research on melatonin receptors in cardiometabolic disorders. Based on the examined melatonin activity scores, the tractability for the melatonin receptors indicates that there is compelling evidence of melatonin discovery in cardiovascular and metabolic disorders, supporting further R&D on cardiometabolic disorders. Differential platform analyses enabled us to screen out targets in different database platforms and connect targets differentially, allowing for the centralization of disease-specific node sets and making the identified targets more selective, thereby increasing the reliability of the identified therapeutic target. When melatonin or its agonists are successful in a triad disorder, they could also be beneficial in other coexistent triad disorders. On the basis of these findings, we propose combining outcome measures with biomarkers from different triad diseases when designing clinical trials for melatonin or agonists for a triad disorder.

In this study, we examined the target prediction for melatonin receptors for a group of interrelated diseases using the following open-access platforms: Europe PMC (data type: text mining), ChEMBL (data type: drugs), Open Targets Genetics Portal (data type: genetic associations), PhenoDigm (data type: animal models), and PheWAS (data type: genetic associations). The investigations on these databases yielded different types and varying levels of evidence for melatonin-disease triad correlations based on genetics, somatic mutations, drugs, pathways, expression, text mining, and animal models. This use case highlights how multiplatform, integrated approaches enable targets such as melatonin receptors to be examined further in experimental trials for druggability in the suggested disease triad. Europe PMC provided the most complex melatonin–disease associations, with all melatonin receptors MT1 and MT2 revealed to be associated with several diseases belonging to the psychosocial-sleep/circadian-cardiometabolic disorders triad, followed by ChEMBL, where MT1 and MT2 were identified as targets. Open Targets Genetics and PheWAS identified the melatonin MT2 gene to be associated with the studied disease triad. Phenodigm captured the similarity between a melatonin receptors MT2 knockout mouse and the investigated human disease. Association scores are given to each link between melatonin as a target and a disease based on how strong the evidence is in different databases. Importantly, these ranking systems, which combine multiple datatypes, may, in some circumstances, be arbitrary, and hence the exact ranks are less relevant; the significance of these score systems resides in the fact that they alert disease experts to targets or pathways that they were previously unaware of [42].

Melatonin target tractability was investigated using melatonin activity scores determined by supervised machine learning. The melatonin activity scores estimate the chance of locating a modulator that effectively interacts with the target or pathway. Melatonin activity levels were investigated in cardiovascular, neuronal, and metabolic experimental tests, as these are the biological systems principally engaged in the psychosocial-sleep/circadian-cardiometabolic disorder triad. In cardiovascular, neuronal, or metabolic experimental assays, we found 546 records with melatonin activity scores greater than 4, actual or predicted. The melatonin activity scores were significantly higher in cardiovascular and neuronal assays in comparison to metabolic studies assays at the cell, tissue, and organism levels. These findings indicate that, while melatonin research and development in sleep/circadian and psychosocial disorders is more advanced, as indicated by melatonin association scores, strong evidence on melatonin discovery in cardiovascular and metabolic disorders, as indicated by melatonin activity scores, supports further R&D on cardiometabolic disorders. Identifying the mechanisms of action of possible drugs and their interactions with other systems are critical phases in drug discovery, research, and development. When considering a new chemical target for a prospective drug development program, it is crucial to obtain an early understanding of whether the compound has been explored in various activity assays [43,44]. The mechanisms underlying melatonin’s positive effects on the triad of psychosocial-sleep/circadian-cardiometabolic disorders are being studied. Melatonin is thought to be a major modulator in the molecular pathways behind the link between anxiety, depression, and circadian/sleep disruptions [35,45]. Melatonin and the circadian clock are fundamental for regulating daily activities of the hypothalamic-pituitary-adrenal and autonomic nervous systems in response to stressors throughout the day [46,47,48]. Melatonin plays an important part in neuroendocrine systems, which are major determinants of the normal 24-h blood pressure and heart rate rhythm [49,50]. Our group’s research has found connections between melatonin and angiotensin that modulate the circadian cardiovascular system on multiple levels, leading us to postulate an angiotensin–melatonin axis [51]. This axis modulates the cardiovascular reactions to stress [52,53,54]. Psychosocial mental indications approved for melatonin or agonists include adults with depression (agomelatine), and autism spectrum disorder (ASD) in children [55]. Melatonin agonists have been approved for the treatment of circadian rhythm sleep-wake disorders, including primary insomnia (melatonin and ramelteon), non-24-h sleep-wake disorder, and Smith–Magenis syndrome (tasimelteon) [56]. Cardiometabolic diseases as potential indications for melatonin or its agonists have been recently reviewed [4].

As we highlighted potential therapeutic indications for melatonin in the psychosocial-sleep/circadian-cardiometabolic disorder triad, this prompted us to outline outcome measures and markers for each category in the triad that could be jointly used in clinical trials on melatonin. Although we do not consider this list exhaustive, we have listed outcome measures that could be used to explore linked morbidities in the disease triad, including measures studied by our team.

Psychosocial Outcomes. The Diagnostic and Statistical Manual of Mental Disorders (DSM) and International Classification of Diseases (ICD) are the two diagnostic classification systems that can be used in hospital, outpatient, and community settings to diagnose psychosocial disorders [57]. Anxiety and depression are most commonly assessed using the Goldberg Anxiety and Depression Scale (GADS) or the Hospital Anxiety and Depression Scale (HADS). Major depressive episodes can be detected using a screener that meets the DSM–5 diagnostic criteria, such as the Clinically Useful Depression Outcome Scale (CUDOS), Quick Inventory of Depressive Symptomatology-Self-Report (QIDS-SR), or Patient Health Questionnaire (PHQ-9) [37,38,58,59]. Burnout is assessed using a variety of Patient-Reported Outcome Measures (PROMs): the Maslach Burnout Inventory (MBI), the Pines’ Burnout Measure (BM), the Psychologist Burnout Inventory (PBI), the OLdenburg Burnout Inventory (OLBI), and the Copenhagen Burnout Inventory (CBI) [60]. The Maslach Burnout Inventory (MBI) assesses three aspects of burnout: emotional exhaustion, depersonalization, and personal accomplishment [61]. Vital exhaustion is most frequently assessed by using the Maastricht Vital Exhaustion Questionnaire, followed by the Short Form-36 [62].

Sleep/Circadian Outcomes. To reliably track sleep and circadian physiology and behavior, objective instruments capable of quantifying sleep and circadian function at point-of-care (p-o-c) settings, such as validated wearable technology, are required [63]. The dim-light melatonin onset (DLMO) assessed in blood or saliva is considered a biomarker for the suprachiasmatic nucleus (SCN) phase [64]. Urinary 6-sulfatoxymelatonin (melatonin’s metabolite) in the first-morning urine determines nighttime suppression of melatonin synthesis [65,66,67,68]. By measuring urinary 6-sulfatoximelatonin, we were able to identify preeclampsia, gestational diabetes [69], and social jetlag [70].

Cardiometabolic Outcomes. Blood pressure variability assessed with ambulatory blood pressure monitoring (ABPM) is advocated by most hypertension guidelines as a validation tool for the traditional wake-time office-based hypertension diagnosis [71,72,73]. Nighttime blood pressure alone, without daytime monitoring, may be a predictor of incident cardiovascular disease events [74], with the strongest connection between 02:00 and 03:00 h, when melatonin production peaks [75]. Our group found algorithms of the night heart rate variability to be associated with sleep apnea severity [76]. Our research has demonstrated that the deep breathing test’s heart rate variability is a reliable method for examining cardiac dysautonomia in conditions related to cardiovascular and psychosocial health [77,78,79]. Angiotensinogen in first-morning urine is associated with a decreased nocturnal melatonin secretion [80] and has been increased in chronic kidney disease [81], preeclampsia, and gestational diabetes [69].

Composite Cardiovascular Risk Scores. Cardiovascular risk scores are calculated by combining clinical, biochemical, anthropometric, behavioral, and lifestyle parameters. SCORE2 and SCORE2-OP are cardiovascular risk scores for the Europe population that incorporate sex-specific and competing risk-adjusted models, including age, smoking status, systolic blood pressure, and total and HDL cholesterol [82,83]. INTERHEART Modifiable Risk Score (IHMRS) includes data on age, sex, status with respect to smoking, diabetes, high blood pressure, family history of heart disease, waist-to-hip ratio, psychosocial factors, diet, and physical activity [84]. The Fuster-BEWAT score (FBS) is a cardiovascular health metric consisting of five modifiable risk factors: blood pressure, exercise, weight, alimentation, and tobacco [85,86]. Life’s Simple 7 Cardiovascular Health (CVH) Metrics are based on smoking, diet, physical activity, body mass index, blood pressure, total cholesterol, and fasting glucose in adults and children [87,88].

### Limitations of the Study

While bioinformatics research utilizing open-access platforms are essential resources for enabling drug development with added value when multiple platforms are investigated, it is necessary to recognize a number of limitations.

The scoring system can appear idiosyncratic between different databases. Furthermore, as enhancements are made and new data sources are introduced, the rank orders can alter considerably between release versions [42]. Therefore, it is important to realize that under-studied diseases are unlikely to provide high-scoring targets due to a lack of available evidence. In such cases, a relatively low-scoring target, such as from the PheWAS platform, may nevertheless be the top-ranked target and possibly a very interesting therapeutic lead. Existing public databases are largely devoid of chemically- and surgically-induced disease models, such as streptozotocin-induced diabetes. Many disease models (particularly in vivo and ex vivo) are only accessible through academics who produced them, and there is currently no adequate database for them. Last but not least, although artificial intelligence and machine learning technologies and algorithms are regarded as having a very high level of precision, they are prone to bias, therefore, they cannot replace experimental investigations in the drug discovery process [89].

These findings point to melatonin’s pleiotropy and druggability, as evidenced by its wide-ranging effects on a variety of currently classified conditions. Future research should clarify the presence of the endogenous mitochondrial melatonergic pathway in cardiomyocytes, endothelial cells, and immune cells. Recent research suggests that circadian-linked pineal melatonin may act to upregulate the mitochondrial melatonergic pathway, with direct consequences for mitochondrial function, including ROS production and the impact this has on ROS-driven miRNAs, and thus on wider gene patterning. Endogenous melatonin regulation in immune cells, including macrophages and microglia [90,91], demonstrates that immune cells shift to an M2-like phenotype via the autocrine effects of NF-kB, allowing melatonin to limit the duration and consequences of immune inflammation. It was recently proposed that variations in the melatonergic pathway in cardiomyocytes may be important regulators of the left ventricular hypertrophy [92]. Because an array of diverse factors, such as tryptophan availability, uptake, and conversion to serotonin, as well as the driving of serotonin down the mitochondrial melatonergic pathway, can all be dynamically regulated in different cell types, such as variations in the availability of different 14-3-3 isoforms [93], many of the effects of circadian and pharmaceutical melatonin remain to be investigated. Such investigations should better clarify the impact of variations in circadian and pharmaceutical melatonin for the seemingly distinct, but interrelated triad of disorders highlighted in this article.

## 4. Materials and Methods

According to the International Union of Basic and Clinical Pharmacology (IUPHAR) receptor nomenclature [94], the melatonin receptors investigated here as drug discovery targets were the MT1 receptor (synonyms: MTNR1A, MEL-1A-R, Melatonin receptor type 1A, Mel1a receptor) and MT2 receptor (synonyms: MTNR1B, Melatonin receptor type 1B, Mel-1B-R, Mel1b receptor, FGQTL2, melatonin receptor 1B variant b, melatonin receptor MEL1B).

### 4.1. Unsupervised and Supervised Machine Learning

To investigate target prediction for melatonin, we examined the target–disease relationships for melatonin receptors in a study that combined searches from multiple databases that apply machine learning processes [89,95] to establish relationships and score them, including Europe PMC (data type: text mining), ChEMBL (data type: drugs), Open Targets Genetics Portal (data type: genetic associations), PhenoDigm (data type: animal models), and PheWAS (data type: genetic associations). The databases are described further in more detail. The target–disease association data were downloaded from json files from the Open Targets data download website (https://platform.opentargets.org/downloads, accessed on 27 August 2022) and then imported into a local database. The diseases with MT1 (MTNR1A) and MT2 (MTNR1B) association records were then retrieved to generate a general association score, evidence count, and the sources used to generate the scores, which were further classified into cardiometabolic disorders, psychosocial disorders, and sleep/circadian disorders. As previously described [96], the associations—direct (by data source) and associations—indirect (by data source) collections were queried to return tables containing gene and disease identifiers, data sources supporting the information, and the amount of evidence for each association and data source. Direct pieces of evidence are disease–target association items that include the exact names of genes and disorders in the body of the evidence. However, if the disease manifests in an ontologically-linked item rather than the item’s body, Open Targets considers the evidence as indirect [97]. The Europe PMC, PhenoDigm, Expression Atlas, Open Targets Genetics site, and ChEMBL database platforms returned findings in response to queries. Once the overall scores and data sources for existing targets and diseases were acquired, particular queries were made to the identified sources to retrieve information specific to each source. The results of queries included both direct and indirect evidence. Information about data sources and how scores are built can be accessed from the Open Targets website (https://platform-docs.opentargets.org/associations, accessed on 11 October 2022).

The potential of melatonin as a tractable target in the management of the disorder triad was investigated by using supervised machine learning to identify activities of melatonin in cardiovascular, neuronal, and metabolic assays at the cell, tissue, and organism levels in a curated ChEMBL database [43]. A curated ChEMBL database was also investigated for melatonin receptor activities in cardiovascular, neuronal, or metabolic experimental assays at the cell, tissue, and organism levels. The ChEMBL database that we used for machine learning and identification of experimental assays is a manually-curated database that collects information from more than 15 million experimental records carried out with 2 million molecules in 1.4 million different phenotypic or target-based assays, which is distinct from the ChEMBL data stored in the Open Targets database that contain information regarding the mechanisms of action of approved drugs or substances submitted for clinical testing [96]. The curated ChEMBL features over 15 million experimental records with normalized scores, which considerably improves the capability of using ChEMBL information, and includes 2 million chemicals and 1.4 million distinct phenotypic or target-based assays (https://doctortarget.com, accessed on 27 August 2022). We previously identified 9k compounds with melatonin potency scores > 4, which corresponds to the intuitive range of activity for a typical pXC50 value by translating the ChEMBL activity values into a value similar to log (potency) in molar scale [4,96]. Given the high correlation between the activities of the MTR1A and MTR1B receptors [4], an average melatonin potency score was employed for supervised machine learning. The evaluation of prediction models proceeded as previously reported [4]. The validation set was split in an 80/20 ratio of training and test sets. The training set was utilized to create random forest classification and regression models. The test set was then run through the model after the real melatonin score and labels were removed. This provided a melatonin prediction based on the 20% of the validation set data that could be compared to the actual values previously deleted, allowing prediction quality evaluation.

### 4.2. Investigated Open-Access Databases

The Europe PMC (EMBL-EBI Europe PubMed Central) enables access to worldwide life science publications and preprints from trusted sources. The Europe PMC data source aims to identify target–disease co-occurrences in the literature and assess the relationship’s confidence. This pipeline uses deep-learning-based named entity recognition (NER) to identify genes/proteins and diseases when mentioned in the text, to later normalize them to the target or disease/phenotype entities in the platform. All co-occurrences of both entity types in the same sentence are considered evidence. In the platform, a piece of Europe PMC evidence results from aggregating all co-occurrences of the same target and disease within the same publication (data type: text mining). Scores are based on weighted document sections, sentence locations, and titles for full-text articles and abstracts, as described in Kafkas et al., 2017 [98]. The aggregated scores of each gene/disease co-occurrence in the publication are further normalized between 0 and 1.

The EMBL-EBI ChEMBL is a manually-curated database of bioactive molecules with drug-like properties, either approved for marketing by the U.S. Food and Drug Administration (FDA) or clinical candidates. ChEMBL also captures information on drug-molecule indications as well as their curated pharmacological target. In the platform, ChEMBL evidence represents any target–disease relationship that can be explained by an approved or clinical candidate drug targeting the gene product and indicated for the disease [99]. Independent studies are treated as individual evidence. To provide additional context, we integrate a machine learning-based analysis of the reasons why a clinical trial has ended earlier than scheduled. This sorts the stop reasons into a set of 17 classes, which include negative, neutral, and positive reasons. This information is available when hovering on the tooltip of the Source column. The 17 classes are Another Study, Business or Administrative, Negative, Study Design, Invalid Reason, Ethical Reason, Insufficient Data, Insufficient Enrolment, Study Staff Moved, Endpoint Met, Regulatory, Logistics or Resources, Safety and Side Effects, No Context, Success, Interim Analysis, and COVID-19 (data type: drugs). ChEMBL evidence scores are based on clinical precedence: 0 for Phase 0, 0.1 for Phase I, 0.2 for Phase II, 0.7 for Phase III, and 1 for Phase IV clinical trials.

The Open Targets Genetics Portal focuses on the identification of trait-causal genes from significant loci in genome-wide association studies (GWAS) [100]. Whereas GWAS identifies significantly associated alleles (lead variants), these variants might not necessarily be the causal (or the only causal) ones. Moreover, causal genes are not necessarily the closest to the lead variant. Due to these reasons, identifying target–disease associations based on GWAS data is extremely challenging. Open Targets Genetics tackles this and other challenges by applying cutting-edge statistical genetics methodologies to large-scale human genetics data. Moreover, Open Targets Genetics uses a machine learning method to identify the most likely causal genes by integrating and summarizing the effect of tag variants based on genetic and functional genomic data. This method is referred to as the Locus2Gene model. Evidence from the Genetics Portal platform (data type: genetic associations) is defined as any significant lead variant with GWAS (*p*-value < 1 × 10^−8^) identified in a study with a predicted causal gene for the given trait with a Locus2gene score greater than 0.05 [100].

The Wellcome Sanger Institute PhenoDigm is an algorithm aimed at prioritizing disease-causing genes based on the phenotype information [101]. By leveraging information from the International Mouse Phenotypes Consortium (IMPC) on mouse knockout phenotypes, PhenoDigm (data type: animal models) aims to systematically map the phenotypes observed in mice to potentially equivalent human diseases. The premise is that, if a gene knockout causes an equivalent phenotype in mice, the human counterpart is likely to be related to the cause of the disease. It uses a semantic approach to map clinical features observed in human and mouse phenotype annotations. The phenotypic effects in mice are then mapped to phenotypes associated with human diseases. Matches are identified, and a similarity score between a mouse model and a human disease is computed. The evidence score indicates the degree of concordance between the mouse and disease phenotypes, as described by Smedley et al. [101].

The PheWAS catalog provides a comprehensive analysis of significantly associated loci across the phenome. To run PheWAS, the R PheWAS package was utilized [102]. The list of phenotypes is derived from electronic medical records (EMRs) represented in the BioVU DNA biobank produced by the Center for Precision Medicine at Vanderbilt University Medical Center. The EMR-based PheWAS uses ICD9 (International Classification of Disease, 9th edition) mapped to EFO (Experimental Factor Ontology) using OLS (Ontology Lookup Service) and Zooma. The evidence of the PheWAS catalog (data type: genetic associations) in the platform is defined as any variant associated with a significant PheWAS trait. Gene burden data include gene–phenotype connections discovered through gene-level association testing with rare variant collapsing analysis. These relationships are the result of combining the effects of all rare variants in a gene into a single burden statistic and regressing the phenotype on the burden statistic to test for the combined effects of all rare variants in that gene. The various collapsing methods provide information about the filters used to pick the set of eligible variations, which are largely based on pathogenicity and population frequency. Evidence scoring is a scaled *p*-value from 0.25 (*p* = 1 × 10^−7^) to 1 (*p* < 1 × 10^−17^) [103].

### 4.3. Data Visualization and Statistical Analysis

Data visualization on target–disease associations was done by graphs created using “igraph” library-based scripts and displayed using the Gephi ForceAtlas algorithm. The resulting data table is attached in the supplementary results. This table has been downloaded in R to build graphs by applying scripts based on the “igraph” library (see code in the Supplementary File S2—R code used to build graphs). The graph information has been stored in graphml format and edited with Gephi software, allowing parallel edges when different data sources agree on the same target–disease association. Graphs have been depicted applying the Gephi ForceAtlas algorithm. The node size has been set to the degree of the node (number of edges or associations) for graphs where different groups of diseases were present. When the graph represented a single disease group, the size was set to the aggregated average score for each node from all association scores connected to that node. The width of the edges is always proportional to the average association score for each specific pair of target–disease nodes.

To determine data normality within each study group, the D’Agostino and Pearson omnibus and Kolmogorov–Smirnov tests (with Dallal–Wilkinson–Lillie for corrected *p*-value) were utilized. Differences in evidence scores (melatonin association and activity scores) between study groups were analyzed using the Kruskal–Wallis nonparametric test, followed by multiple comparisons of each group’s mean rank using Dunn’s multiple testing correction for statistical hypothesis testing. GraphPad Prism version 8.1.2 for Mac OS X, GraphPad Software, La Jolla, California, USA, www.graphpad.com, accessed on 27 August 2022, was used for all statistical analyses.

## 5. Conclusions

This study provides the target prediction and tractability of melatonin receptors for the proposed psychosocial-sleep/circadian-cardiometabolic disorder triad as a cluster of diseases with interoperable pathologies. These findings point to melatonin’s pleiotropy and druggability, as evidenced by its effects on several currently classified conditions. Future research on melatonin’s recently discovered intracellular roles, such as the mitochondrial melatonergic pathway, which is emerging as a potent regulator of mitochondrial function, will help better to define relevant pathophysiological processes and treatment targets for melatonin. Whereas each database could only show melatonin receptor–disease associations for one receptor type or a subset of disorders, a multiplatform analysis provided an integrative assessment of the target–disease investigations. We envisage combining outcome measures with biomarkers from various triad diseases when developing clinical trials for melatonin or agonists for a triad disorder. Last but not least, these data may lead to clinical studies on a more regulated prescription of this hormone, as opposed to its consumption as a dietary supplement.

## Data Availability

Datasets analyzed during the study are publicly available.

## Supplementary Materials

The following supporting information can be downloaded at: https://www.mdpi.com/article/10.3390/ijms24010860/s1.
