# Supplementary material for: Multiplatform-Integrated Identification of Melatonin Targets for a Triad of Psychosocial-Sleep/Circadian-Cardiometabolic Disorders
Source: Int J Mol Sci. 2023 Jan 3;24(1):860. doi: 10.3390/ijms24010860 (PMC9821171; doi:10.3390/ijms24010860)
Supplement: Supplementary file 1 [file ijms-24-00860-s001.zip › S2 -R code used to build graphs.pdf]

**R code used to build graphs:**

```
#install.packages("igraph")
library(igraph)
library(ggplot2)
library(dplyr)
library(data.table)

myFile<-read.csv(file="//myPath/melatoninDiseasesForGraph.csv") #this is the XL table reported
colnames(myFile)
colnames(myFile)[1]<-"datasourceId"
myFileNodes<-myFile[, c("targetSymbol", "diseaseLabel")]
library(dplyr)
myFile<-select(myFile, -c("targetSymbol", "diseaseLabel"))
myFile<-cbind(myFileNodes, myFile)
rm(myFileNodes)

myNodes<-unique(union(myFile$targetSymbol, myFile$diseaseLabel))

g <- graph.data.frame(myFile, directed=FALSE, vertices=myNodes)
plot(g)
write.graph(g, "000.graphml", format="graphml")

library(tidyr)
targetDetails<-pivot_wider(myFile, id_cols = c(targetSymbol, targetName),
                names_from = diseaseGroup,
                 values_from = c(datasourceHarmonicScore), values_fn = mean,
                 values_fill = 0,
                names_prefix = 'scoreFor')
targetDetails2<-pivot_wider(myFile, id_cols = targetSymbol,
                names_from = diseaseGroup,
                values_from = c(datasourceEvidenceCount), values_fn = sum,
                values_fill = 0,
                names_prefix = 'EvidencesFor')

targetDetails<-merge(targetDetails, targetDetails2, by = 'targetSymbol')
rm(targetDetails2)

myFile$diseaseType<-myFile$diseaseGroup
diseaseDetails<-pivot_wider(myFile, id_cols = c(diseaseLabel, diseaseType),
                names_from = diseaseGroup,
                values_from = c(datasourceHarmonicScore), values_fn = mean,
                values_fill = 0,
                names_prefix = 'scoreFor')
diseaseDetails2<-pivot_wider(myFile, id_cols = diseaseLabel,
                 names_from = diseaseGroup,
                 values_from = c(datasourceEvidenceCount), values_fn = sum,
                 values_fill = 0,
```

```
                        names_prefix = 'EvidencesFor')

diseaseDetails<-merge(diseaseDetails, diseaseDetails2, by = 'diseaseLabel')
rm(diseaseDetails2)

diseaseDetails$targetName<-'disease'
diseaseDetails$nodeType<-'disease'
targetDetails$diseaseType<-'gene'
targetDetails$nodeType<-'gene'

colnames(diseaseDetails)[1]<-"nodeName"
colnames(targetDetails)[1]<-"nodeName"
myNodeLabels<-rbind(targetDetails, diseaseDetails)

library(data.table)
myNodes<-fread("000Nodes.csv")
myNodes<-merge(myNodes, myNodeLabels, by.x = 'v_name', by.y = 'nodeName' )
fwrite(myNodes, "000Nodes.csv")
```
